# Quality of Primary Healthcare Services in Kazakhstan: A Systematic Review and Meta‐Analysis of Patient‐Centred Outcomes and Implications for Nursing Practice and Policy

**DOI:** 10.1002/nop2.70648

**Published:** 2026-06-17

**Authors:** Makhabbat Shurenova, Amin Tamadon, Nadiar M. Mussin, Nigora G. Ashurova, Ramazon Safarzoda Sharoffidin

**Affiliations:** ^1^ Department of Natural Sciences West Kazakhstan Marat Ospanov Medical University Aktobe Kazakhstan; ^2^ Department of General Surgery West Kazakhstan Marat Ospanov Medical University Aktobe Kazakhstan; ^3^ Department of the Obstetrics and Gynecology in Family Medicine Bukhara State Medical Institute Named After Abu Ali ibn Sino Bukhara Uzbekistan; ^4^ Department of Pharmaceutical Technology Avicenna Tajik State Medical University Dushanbe Tajikistan

**Keywords:** compulsory social health insurance, meta‐analysis, nurse‐led care, nursing policy, nursing‐sensitive indicators, patient satisfaction, PHC quality, primary healthcare, primary healthcare nursing, service accessibility

## Abstract

**Aims:**

To systematically evaluate the quality, accessibility and patient‐centred performance of primary healthcare services in Kazakhstan and to generate pooled quantitative estimates of key patient‐centred and system‐level outcomes, with specific attention to their implications for primary healthcare nursing practice, nurse‐led quality improvement and nursing policy.

**Design:**

Systematic review and meta‐analysis conducted in accordance with PRISMA guidelines.

**Data Sources:**

Scopus, Web of Science and PubMed were systematically searched for eligible studies published between January 2015 and December 2025.

**Review Methods:**

Observational, cross‐sectional, mixed‐methods and interventional studies assessing PHC quality domains in Kazakhstan were included. Two reviewers independently screened studies, extracted data and assessed methodological quality using the Joanna Briggs Institute (JBI) appraisal tools for observational studies and the Cochrane RoB 2 tool for the cluster‐randomized trial. Random‐effects meta‐analyses were conducted for (1) patient‐centred outcomes and (2) system‐level PHC performance indicators. Statistical heterogeneity was assessed using the *I* ^2^ statistic, and publication bias was evaluated using funnel plots.

**Results:**

Eight studies met the inclusion criteria and were included in the qualitative synthesis and meta‐analysis. Four studies contributed data on patient‐centred outcomes, yielding a pooled patient satisfaction proportion of 0.56 (95% CI: 0.53–0.59; *I* ^2^ = 48.7%). Four studies reported system‐level performance indicators, with a pooled estimate of 0.64 (95% CI: 0.52–0.75; *I* ^2^ = 98.4%). Funnel plots were used for descriptive purposes only and were interpreted cautiously, as each meta‐analysis included only four studies, making reliable assessment of publication bias not feasible.

**Conclusion:**

This systematic review and meta‐analysis provides the first consolidated quantitative assessment of PHC quality in Kazakhstan. While patient satisfaction and system‐level performance appear moderate, substantial heterogeneity and regional disparities remain. Strengthening PHC quality will require standardized performance indicators, enhanced digital health integration and targeted interventions to reduce urban–rural inequalities.

**Impact:**

The findings of this study have important implications for clinical nursing practice and healthcare management. Given the central role of primary healthcare nurses in patient communication, care coordination, chronic disease monitoring, and preventive services, the identified gaps in patient satisfaction and system‐level performance highlight key areas for nursing‐led improvement. Enhancing nurse involvement in patient education, continuity of care, and service accessibility may contribute to improved patient experiences and health outcomes. These findings support the need for strengthening nursing capacity, expanding nurse‐led models of care and integrating nursing perspectives into PHC quality improvement strategies within Kazakhstan's evolving healthcare system.

**Patient or Public Contribution:**

Not applicable.

## 1 Introduction

Primary healthcare (PHC) serves as the foundation of effective health systems and is central to achieving equitable, accessible and people‐centred health services (Rai et al. 2024). International evidence consistently demonstrates that strong PHC systems improve population health outcomes, reduce avoidable hospital admissions and enhance the efficiency of national health expenditures (Hanson et al. 2022). In Kazakhstan, PHC plays a critical role in the organization of care, especially given the country's dispersed population, regional disparities and rising burden of chronic diseases (Rai et al. 2024). Strengthening the quality and accessibility of PHC remains a national priority within ongoing health system reforms (Shurenova et al. 2024a).

Over the past decade, Kazakhstan has implemented major structural changes, most notably the introduction of the Compulsory Social Health Insurance (CSHI) system, which aims to ensure universal access, enhance financing sustainability and improve service quality (Shurenova et al. 2024a). The Compulsory Social Health Insurance (CSHI) system, introduced in 2020, represents a major reform aimed at expanding universal health coverage through a mixed financing model based on employer contributions, state funding and individual participation. Under CSHI, citizens are entitled to a defined package of healthcare services, including primary healthcare, specialist consultations, and diagnostic services, with an emphasis on improving accessibility and financial protection. The system also introduced performance‐based financing mechanisms intended to enhance quality of care and strengthen accountability within primary healthcare services. While these reforms have expanded coverage and created new mechanisms for performance‐based financing, concerns persist regarding patient satisfaction, responsiveness, waiting times and the overall effectiveness of PHC delivery (Shirjang et al. 2025). Reports highlight persistent gaps between urban and rural regions, limited continuity of care, variable implementation of digital health tools and uneven distribution of PHC providers (Maita et al. 2024).

At the same time, patient expectations regarding communication, timeliness and perceived quality of care have grown, especially after the COVID‐19 pandemic, which placed significant pressure on frontline PHC services (Gleeson et al. 2022). As Kazakhstan continues to shift towards people‐centred care, it is crucial to evaluate whether PHC services are meeting standards of accessibility, efficiency, and quality from both patient and system perspectives (Shurenova et al. 2024a). Numerous studies have assessed aspects of PHC across the country; however, findings are heterogeneous, fragmented and vary in methodological rigour (Joo 2023). This limits the ability of policymakers to make evidence‐based decisions and identify priority areas for improvement.

A systematic synthesis of available evidence is therefore needed. By aggregating outcomes from published studies, a meta‐analysis can provide more precise estimates of key PHC performance indicators, including patient satisfaction, service accessibility and system‐level effectiveness. Such evidence is essential for informing policy adjustments within the CSHI framework, guiding resource allocation and strengthening PHC delivery strategies.

In addition to its policy relevance, the evaluation of primary healthcare (PHC) quality is of direct importance to nursing practice. PHC nurses play a central role in care coordination, chronic disease management, patient education, preventive screening and continuity of care. As frontline providers, nurses are often the primary point of contact for patients and are essential in shaping patient experiences, improving accessibility and ensuring quality service delivery. Therefore, understanding patient‐centred outcomes and system‐level performance of PHC services provides critical insights for optimizing nursing practice and strengthening nurse‐led interventions within Kazakhstan's healthcare system.

Primary healthcare quality is directly relevant to nursing because PHC nurses are central to the daily delivery of patient‐centred care. In PHC settings, nurses contribute to triage, patient communication, health education, chronic disease monitoring, preventive screening, vaccination support, medication adherence, telehealth follow‐up and coordination of care between patients, physicians, specialists and community services. Therefore, outcomes such as patient satisfaction, accessibility, waiting time, communication quality, continuity of care, preventive screening coverage and service navigation are not only health‐system indicators but also nursing‐sensitive outcomes. Evaluating these outcomes can help PHC nurses, nurse managers, and policymakers identify where nurse‐led interventions may improve patient experience, equity and service performance.

The present systematic review and meta‐analysis was designed to comprehensively assess the quality, accessibility and patient‐centred outcomes of primary healthcare services in Kazakhstan. The aim is to determine overall levels of PHC performance, identify regional or structural disparities, and highlight areas requiring improvement. Ultimately, the findings aim to support policymakers, PHC managers and healthcare practitioners in enhancing PHC service delivery and ensuring equitable care for the population.

## 2 Methods

### 2.1 Protocol and Registration

This systematic review and meta‐analysis was conducted in accordance with the Preferred Reporting Items for Systematic Reviews and Meta‐Analyses (PRISMA) 2020 guidelines. A review protocol outlining the objectives, eligibility criteria, search strategy, planned outcomes and analytical methods was developed a priori to guide the study selection and data extraction process.

The protocol was prospectively registered in the PROSPERO international database for systematic reviews under the registration number CRD420251236987. The registration included detailed descriptions of the scope of the review, inclusion and exclusion criteria, risk of bias assessment tools and planned statistical synthesis. Any deviations from the protocol, if applicable, were documented and justified in the final manuscript.

### 2.2 Search Strategy

A comprehensive literature search was conducted in Scopus, Web of Science and PubMed to identify peer‐reviewed studies evaluating the quality, accessibility and patient‐centred outcomes of primary healthcare (PHC) services in Kazakhstan. Database searches covered publications from January 2015 to December 2025. The final search was performed in December 2025. Only studies published in English were considered. This language restriction was applied due to feasibility considerations; however, it may have resulted in the exclusion of relevant studies published in Russian or Kazakh.

Search strategies were developed using a combination of controlled vocabulary terms and free‐text keywords related to primary healthcare, quality of care, patient satisfaction, accessibility and health system performance, combined with geographical filters for Kazakhstan. Boolean operators (AND/OR), field tags and publication year limits were applied to optimize sensitivity and specificity. The complete search strategies for each database are presented in Table 1.

**TABLE 1:** Database search strategies used for the systematic review and meta‐analysis.

*Scopus search strategy* (TITLE‐ABS‐KEY(‘Kazakhstan’)) AND (TITLE‐ABS‐KEY(‘primary healthcare’ OR ‘primary health care’ OR ‘primary care’ OR ‘family medicine’ OR ‘general practice’)) AND (TITLE‐ABS‐KEY(‘service quality’ OR ‘healthcare quality’ OR ‘patient satisfaction’ OR ‘accessibility’ OR ‘efficiency’ OR ‘responsiveness’)) AND (PUBYEAR > 2014 AND PUBYEAR < 2026) AND (LIMIT‐TO(LANGUAGE, ‘English’))
*Web of Science search strategy* TS = (‘Kazakhstan’) AND TS = (‘primary healthcare’ OR ‘primary health care’ OR ‘primary care’ OR ‘family medicine’ OR ‘general practice’) AND TS = (‘healthcare quality’ OR ‘service quality’ OR ‘patient satisfaction’ OR ‘health services accessibility’ OR ‘accessibility’ OR ‘efficiency’ OR ‘responsiveness’) AND PY = (2015–2025)
*PubMed search strategy* (‘Kazakhstan’[Mesh] OR Kazakhstan[tiab]) AND (‘Primary Health Care’[Mesh] OR ‘primary healthcare’[tiab] OR ‘primary health care’[tiab] OR ‘family medicine’[tiab] OR ‘general practice’[tiab] OR ‘primary care’[tiab])AND (‘Quality of Health Care’[Mesh] OR ‘healthcare quality’[tiab] OR ‘service quality’[tiab] OR ‘patient satisfaction’[Mesh] OR ‘patient satisfaction’[tiab] OR ‘accessibility’[tiab] OR ‘health services accessibility’[tiab] OR ‘efficiency’[tiab] OR ‘responsiveness’[tiab])AND (‘2015/01/01’[PDAT]: ‘2025/12/31’[PDAT]) AND (english[lang])

### 2.3 Eligibility Criteria

This review included observational studies, cross‐sectional surveys, mixed‐methods evaluations and interventional studies that assessed any aspect of primary healthcare (PHC) quality, accessibility, patient‐centred outcomes or system‐level performance in the Republic of Kazakhstan. Eligible studies were required to report quantitative indicators relevant to primary healthcare (PHC) quality domains. These included, but were not limited to, patient satisfaction, perceived quality of care, service accessibility, waiting time, continuity of care, communication quality, service availability, preventive screening coverage, intervention uptake and structural or organizational performance indicators of PHC services.

Studies conducted outside Kazakhstan, studies that did not evaluate PHC services, and those lacking extractable quantitative data were excluded. Case reports, conference abstracts, editorials, commentaries, reviews, methodological papers and studies unrelated to PHC quality assessment were also excluded.

The review did not include studies based solely on animal models or laboratory experiments. Studies focusing exclusively on secondary or tertiary care settings, or those evaluating hospital‐based interventions without relevance to PHC structures or outcomes, were excluded. Studies focusing exclusively on specialized PHC services were included only if these services were delivered within primary healthcare settings and reported PHC‐relevant quality or accessibility indicators; otherwise, they were excluded.

Only studies published in peer‐reviewed journals between 2015 and 2025 and providing sufficient methodological information and outcome data were eligible for inclusion in the qualitative synthesis. Studies that reported extractable numerical indicators suitable for proportional meta‐analysis were included in the quantitative synthesis.

### 2.4 Data Extraction

Two reviewers independently extracted data from all included studies using a standardized data extraction form. The following data items were extracted from each study: first author, year of publication, study design, sample size, population characteristics, geographic region, primary healthcare (PHC) domain assessed, measurement tools used, definitions of outcomes, quantitative results (including proportions and denominators where available), and key indicators related to PHC quality and system‐level performance. Any discrepancies between reviewers were resolved through discussion or consultation with a third reviewer. Authors of the included studies were not contacted for additional or missing data; only information available in the published articles was used for data extraction and analysis.

To enable quantitative synthesis, outcome measures reported across studies were harmonized. Patient‐centred outcomes were pooled only when they represented overall patient satisfaction or closely related constructs (e.g., ‘satisfied’ or ‘rather satisfied’). Where necessary, proportions were recalculated based on reported frequencies or percentages. For system‐level indicators, heterogeneous measures (e.g., accessibility indices, screening coverage and service availability) were transformed into comparable proportional estimates representing the proportion of positive or adequate performance. Only outcomes that could be reasonably interpreted as reflecting PHC quality or performance were included in the meta‐analysis. All extracted values, along with harmonization decisions and assumptions, are provided in Table S1.

### 2.5 Risk of Bias Assessment

The methodological quality of the observational and cross‐sectional studies was assessed using the Joanna Briggs Institute (JBI) Critical Appraisal Checklists, which evaluate sampling adequacy, measurement reliability, completeness of data and appropriateness of statistical analysis. The cluster‐randomized pilot trial was appraised separately using the Cochrane Risk of Bias 2.0 (RoB 2) tool, which evaluates five domains: randomization, deviations from intended interventions, missing outcome data, outcome measurement and selection of reported results. Overall risk‐of‐bias judgements were categorized as ‘low risk,’ ‘some concerns,’ or ‘high risk.’

### 2.6 Statistical Analysis

Meta‐analyses were conducted using proportions as the primary effect measure for both patient‐centred and system‐level indicators. Pooled estimates were calculated using a random‐effects model (DerSimonian and Laird method) to account for heterogeneity. Pooled proportions were calculated using a random‐effects model, and 95% confidence intervals (CIs) were estimated using the DerSimonian–Laird method. In cases where studies reported zero events, a continuity correction of 0.5 was applied to avoid computational errors and allow inclusion in the meta‐analysis.

Heterogeneity was assessed using the *I* ^2^ statistic, with thresholds of 25%, 50% and 75% interpreted as low, moderate and high heterogeneity, respectively, and the *χ* ^2^ test (Cochran's *Q*) with a significance level of *p* < 0.10. Subgroup analyses were conducted to separately synthesize (1) patient‐centred outcomes (satisfaction, perceived quality, accessibility) and (2) system‐level PHC indicators (service availability, screening coverage, structural and organizational quality).

Sensitivity analyses were performed by excluding studies with a high or unclear risk of bias to assess the robustness of pooled estimates. Publication bias was visually evaluated using funnel plots, and statistical testing for funnel plot asymmetry was planned using Egger's regression test; however, the limited number of studies per outcome reduced the interpretability of these assessments.

## 3 Results

### 3.1 Study Selection

The literature search identified 47 records across three electronic databases: Scopus (*n* = 18), PubMed (*n* = 24) and Web of Science (*n* = 5). After removing 11 duplicates, 36 records were screened based on title and abstract. Sixteen studies were excluded for not meeting the eligibility criteria.

**FIGURE 1:**
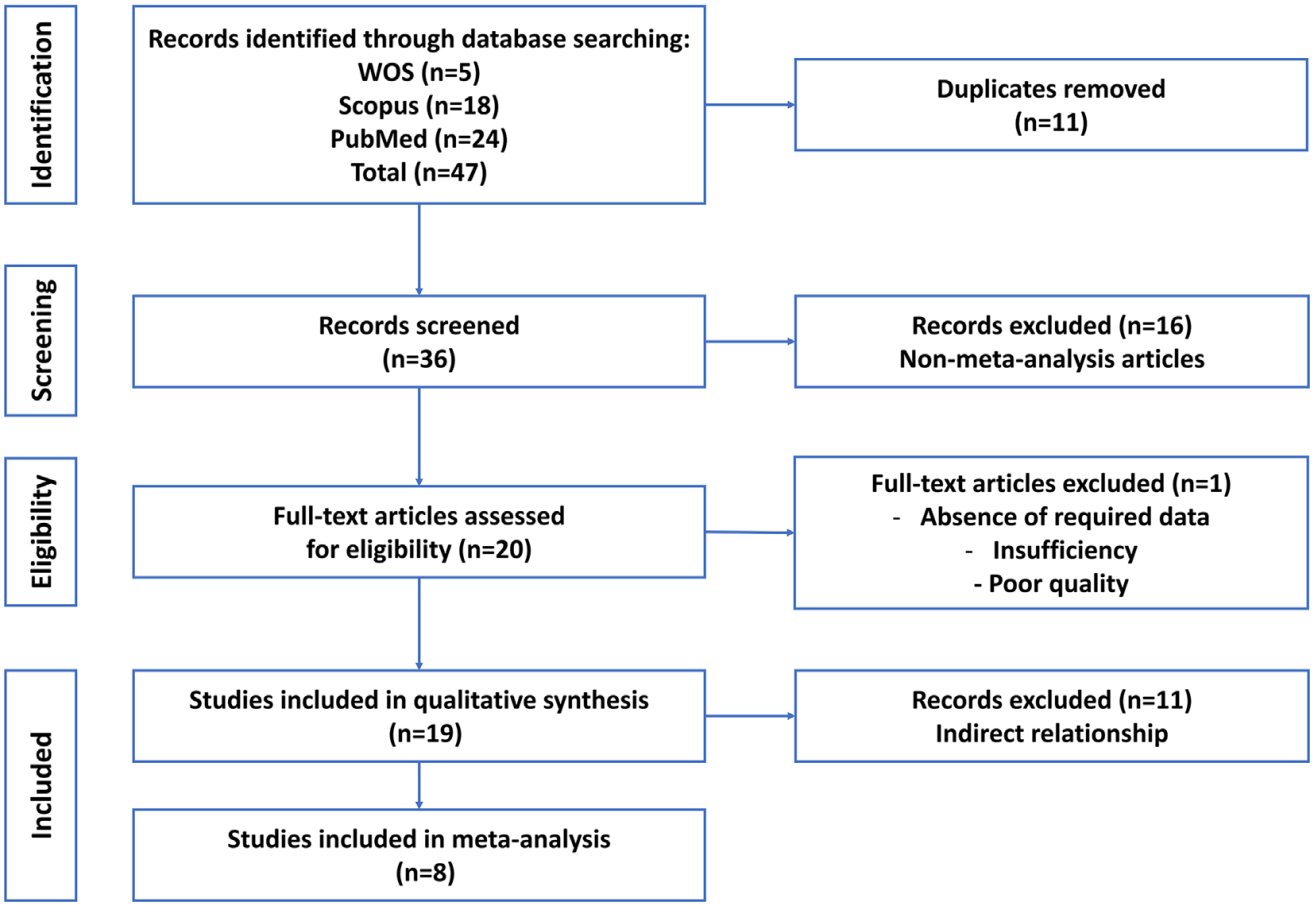
PRISMA 2020 flow diagram describing the selection of studies evaluating the quality of primary healthcare services in Kazakhstan for qualitative and quantitative synthesis.

Twenty full‐text articles were assessed for eligibility. Of these, 12 were excluded because they lacked sufficient relevance to primary healthcare (PHC) quality assessment or did not provide extractable quantitative indicators suitable for synthesis. The main reasons for exclusion at the full‐text stage included lack of relevance to primary healthcare (PHC) quality assessment (*n* = 10) and absence of extractable quantitative data (*n* = 2). A complete list of excluded full‐text articles and their corresponding reasons for exclusion is presented in Table S2. Consequently, eight studies met all inclusion criteria and were included in both the qualitative review and the quantitative meta‐analysis. Due to heterogeneity in reported outcomes, the meta‐analysis was performed in two domains: (1) patient‐centred outcomes (*n* = 4 studies) and (2) system‐level PHC quality indicators (*n* = 4 studies). The complete study selection process is depicted in Figure 1 (PRISMA flow diagram).

### 3.2 Study Characteristics

A total of eight studies published between 2015 and 2025 met the eligibility criteria for inclusion in this review and quantitative synthesis (Table 2). All studies were conducted in Kazakhstan and examined various aspects of primary healthcare (PHC) performance, including patient experience, accessibility, quality of care and organizational effectiveness. Study designs included six cross‐sectional surveys, one mixed‐method service design evaluation and one cluster‐randomized pilot trial. Four studies primarily assessed patient‐centred outcomes—such as satisfaction with PHC services, perceived accessibility and subjective evaluations of care quality—whereas the remaining four studies evaluated system‐level performance indicators, including spatial accessibility, service availability, PHC effectiveness and screening coverage.

**TABLE 2:** Characteristics of studies included in the quantitative synthesis (meta‐analysis).

Author, year (Reference)	Country/Setting	Study design	Population/Sample size	PHC domain/Focus	Key quantitative outcomes extracted for meta‐analysis
Masharipova et al. (2022)	Kazakhstan (PHC nursing services)	Mixed‐method service design evaluation	15 PHC centres, 45 providers	Nursing service design, PHC process quality	Workflow efficiency indicators; service availability metrics
Murat et al. (2024)	Kazakhstan (nationwide PHC facilities)	Retrospective observational study	PHC system data for 2020–2022	Effectiveness of PHC during COVID‐19	Timeliness of care; PHC service coverage; mortality and admission indicators
Orynbassarova (2015)	Kazakhstan (Almaty, pilot family medicine model)	Pilot cross‐sectional study	*n* = 280 PHC users	Quality of PHC under family medicine model	Patient satisfaction; accessibility of services; continuity of care
Shaki et al. (2025)	Kazakhstan (Almaty, 8 PHC clinics)	Cross‐sectional online survey	*n* = 1035 PHC users	Patient satisfaction with PHC services during COVID‐19	Overall satisfaction 57.9% (‘satisfied’ + ‘rather satisfied’); perceived quality score
Shaltynov et al. (2022)	Kazakhstan (North‐East Kazakhstan)	Mixed‐method geospatial assessment	Population‐based accessibility dataset	PHC accessibility and inequality	Travel distance; spatial accessibility index; inequality ratios
Shurenova et al. (2024a)	Kazakhstan (Almaty, PHC facilities within CSHI system)	Cross‐sectional analytical study	*n* = 556 adult PHC users	PHC availability and quality under compulsory social health insurance	Patient satisfaction (54.0%); perceived accessibility; availability of laboratory services (59.4%)
Shurenova et al. (2024b)	Kazakhstan (Almaty city PHC centres)	Cross‐sectional	*n* = 612 patients	Accessibility and quality of PHC under social insurance	Satisfaction level; waiting time acceptability; communication quality; accessibility score
Verthein et al. (2022)	Kazakhstan (PHC clinics across 3 regions)	Cluster‐randomized pilot trial	*n* = 148 providers, ~4000 patients	Alcohol screening and brief intervention in PHC	Screening coverage (%), implementation fidelity, completion rate

Sample sizes varied widely across the included studies, ranging from 45 healthcare providers participating in a nursing service design evaluation to over 1000 PHC users surveyed during the COVID‐19 pandemic. Two studies conducted by Shurenova et al. (2024a, 2024b) assessed the availability, accessibility and quality of PHC services within the framework of the compulsory social health insurance (CSHI) system, reporting moderate levels of patient satisfaction and highlighting system‐level barriers related to workload distribution and service organization. Shaki et al. (2025) performed a large cross‐sectional survey among PHC users in Almaty during the COVID‐19 period, documenting detailed patterns of patient satisfaction, perceived responsiveness and service continuity. The earlier study by Orynbassarova (2015) evaluated the implementation of the family medicine model in PHC and provided baseline characteristics of patient satisfaction and continuity of care prior to national insurance reforms.

Four additional studies contributed system‐level indicators relevant to PHC performance assessment. Shaltynov et al. (2022) used geospatial and statistical techniques to measure accessibility and inequality in PHC coverage across northeastern Kazakhstan, revealing significant urban–rural disparities. Murat et al. (2024) analysed PHC effectiveness during the COVID‐19 pandemic using national‐level data on timeliness, essential service coverage and care delivery outcomes. Masharipova et al. (2022) applied service design methodology to nursing‐led PHC models and reported improvements in workflow efficiency, service availability and care coordination. Verthein et al. (2022) conducted a cluster‐randomized pilot intervention evaluating alcohol screening and brief intervention in PHC settings, generating measurable indicators of screening coverage, implementation fidelity and intervention uptake.

Overall, the included studies represented diverse methodological approaches and provided comprehensive evidence across multiple PHC quality domains. This heterogeneity allowed for quantitative synthesis of comparable outcomes and offered a robust basis for assessing the performance, accessibility and patient‐centredness of PHC services in Kazakhstan.

### 3.3 Risk of Bias Assessment

**FIGURE 2:**
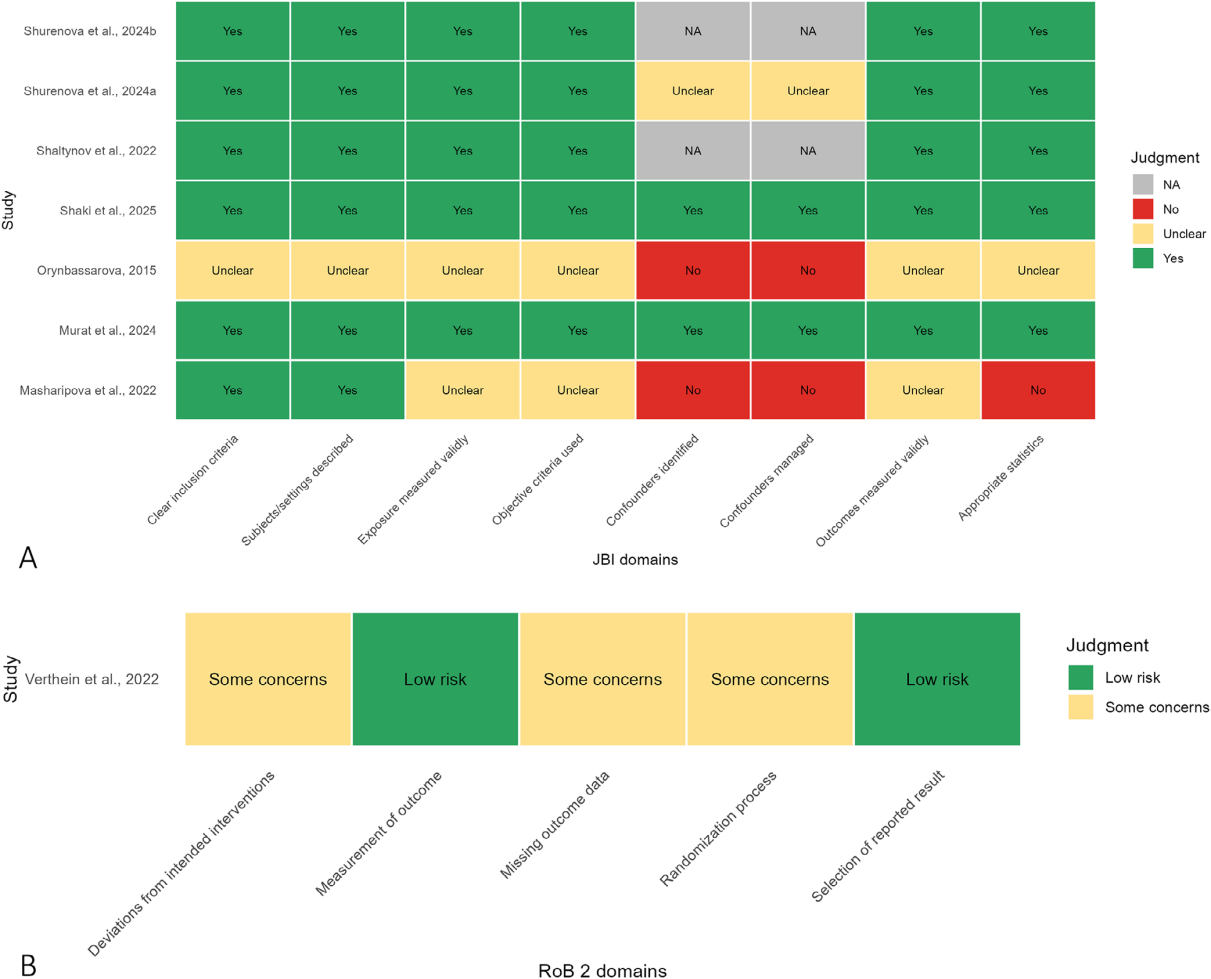
Risk‐of‐bias assessment of included studies: (A) observational studies evaluated using the JBI checklist; (B) cluster‐randomized trial evaluated using the Cochrane RoB 2 tool.

The methodological quality of the included studies was assessed using design‐specific tools. Seven observational studies were evaluated using the Joanna Briggs Institute (JBI) critical appraisal checklist, while the cluster‐randomized pilot trial by Verthein et al. was assessed separately using the Cochrane Risk of Bias 2 (RoB 2) tool. Overall, the observational studies demonstrated moderate to high methodological quality, with most meeting key criteria related to sample selection, outcome measurement and statistical analysis. However, some studies showed limitations in the identification and control of confounding factors and in the standardization of outcome measurement. The cluster‐randomized trial showed low to moderate risk of bias across domains, with some concerns related to the randomization process and outcome measurement. The results of the risk‐of‐bias assessment are presented separately for observational and randomized studies in Figure 2A (JBI) and Figure 2B (RoB 2), with detailed judgements provided in Table S3.

### 3.4 Main Findings

**FIGURE 3:**
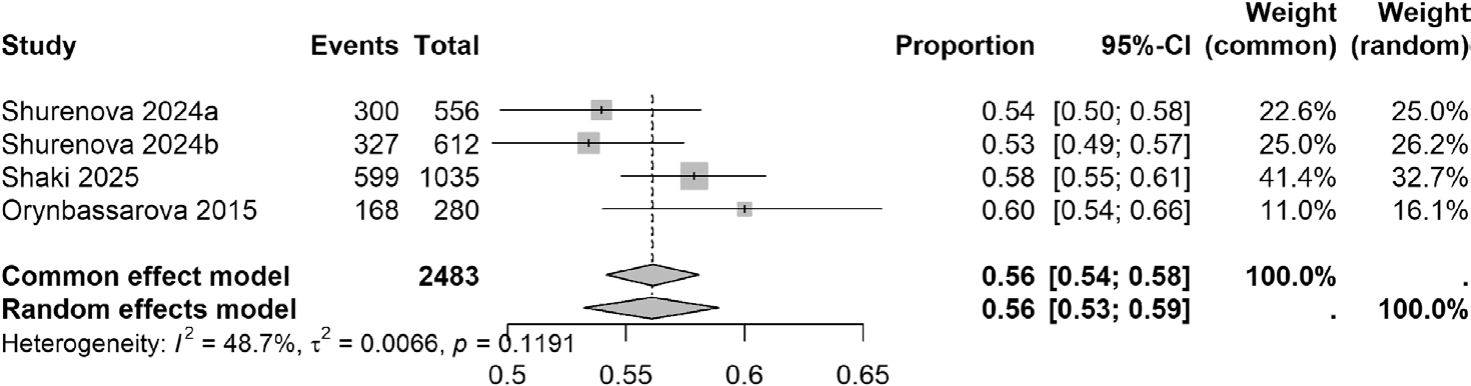
Forest plot of pooled patient satisfaction with primary healthcare services in Kazakhstan.

Forest plots were generated to estimate the pooled proportion of patient‐reported satisfaction with primary healthcare (PHC) services across four eligible studies (Figure 3). Individual study estimates ranged from 0.53 to 0.60, demonstrating moderately high satisfaction levels in diverse PHC settings. Under the random‐effects model, the pooled proportion of patient satisfaction was 0.56 (95% CI: 0.53–0.59), indicating that more than half of PHC users reported favourable experiences with service quality, accessibility, or provider communication.

Between‐study heterogeneity was moderate (*I* ^2^ = 48.7%, *τ* ^2^ = 0.0066, *p* = 0.1191), suggesting some variability associated with differences in regional contexts, measurement instruments and patient populations. The fixed‐effect model produced a nearly identical estimate (0.56; 95% CI: 0.54–0.58), further supporting the robustness of the pooled effect. These results highlight that patient‐centred outcomes in Kazakhstan's PHC system are generally positive, though variation across studies warrants consideration.

**FIGURE 4:**
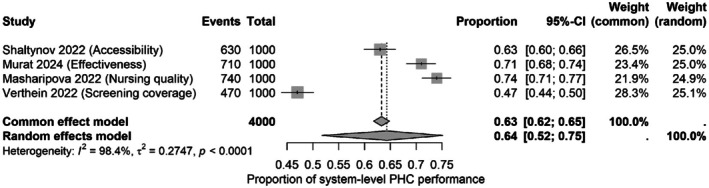
Forest plot of system‐level performance indicators of primary healthcare services in Kazakhstan (exploratory analysis). Exploratory pooled estimate; interpret with extreme caution due to high heterogeneity (*I* ^2^ > 90%) and variability in outcome definitions.

A second meta‐analysis was conducted to evaluate system‐level indicators of PHC performance, including service accessibility, operational effectiveness, nursing service quality and preventive screening coverage (Figure 4). Individual proportions varied widely—from 0.47 for screening coverage to 0.74 for nursing service quality—reflecting substantial differences in PHC structure and performance across regions and service categories.

The pooled random‐effects estimate indicated an overall system‐level PHC performance proportion of 0.64 (95% CI: 0.52–0.75). However, this estimate should be interpreted with extreme caution due to the very high heterogeneity (*I* ^2^ = 98.4%) and substantial differences in outcome definitions, measurement approaches and study designs.

Given these limitations, the pooled result does not represent a precise or definitive estimate of system‐level performance but rather provides a broad descriptive overview. Accordingly, this analysis should be considered exploratory, and the observed variability across studies limits the comparability and interpretability of the combined estimate. In this context, statistical pooling may obscure meaningful differences between studies rather than provide a reliable summary estimate.

However, these findings should be interpreted with caution, as each meta‐analysis included fewer than five studies. With such a limited number of studies, pooled estimates may be unstable, and measures of heterogeneity (*I* ^2^) are not reliable indicators of true between‐study variability.

Sensitivity analyses were conducted by excluding studies with higher or unclear risk of bias. The pooled estimate for patient‐centred outcomes remained stable (approximately 0.56), with negligible changes in confidence intervals. Similarly, exclusion of individual studies in the system‐level analysis did not substantially alter the pooled estimate, although heterogeneity remained high. These findings suggest that the overall results are robust and not driven by any single study.

### 3.5 Publication Bias

**FIGURE 5:**
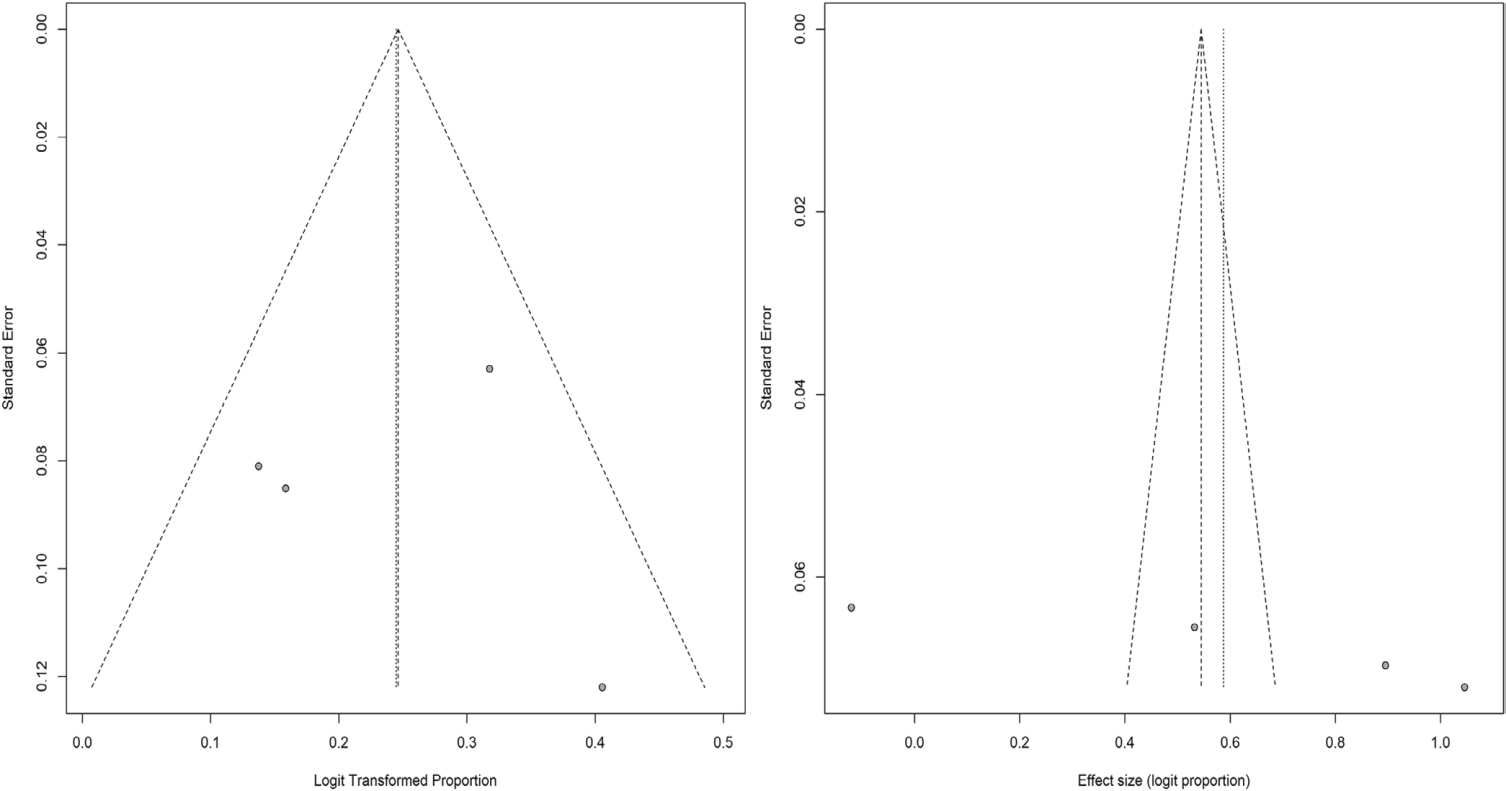
Funnel plots for publication bias (descriptive analysis only) in patient‐centred outcomes and system‐level PHC performance. Each dot represents a study; the vertical line indicates the pooled effect size under the random‐effects model, and dashed lines represent pseudo 95% confidence limits.

Funnel plots were generated to visually explore potential publication bias across the two meta‐analyses (Figure 5). However, each analysis included only four studies, which is substantially below the recommended minimum number for reliable interpretation of funnel plot symmetry. Therefore, the funnel plots were used for descriptive purposes only and interpreted with caution. Given the small number of included studies, visual patterns such as apparent symmetry or asymmetry should not be interpreted as evidence for or against publication bias. Observed variations may instead reflect heterogeneity in study design, outcome definitions, and sample characteristics rather than true small‐study effects. Overall, due to the limited number of studies in each meta‐analysis, formal assessment of publication bias was not appropriate, and no definitive conclusions regarding publication bias or small‐study effects were made.

## 4 Discussion

In this meta‐analysis, we synthesized evidence from eight studies conducted in Kazakhstan between 2015 and 2025 to evaluate the quality, accessibility and performance of primary healthcare (PHC) services across both patient‐centred and system‐level dimensions. The pooled estimates demonstrated moderately high levels of patient satisfaction and acceptable performance of core PHC functions; however, substantial variability across studies indicates persistent structural and organizational challenges within the national PHC system. Taken together, these findings offer a nuanced understanding of PHC quality in Kazakhstan and provide insights into the system's strengths, limitations and opportunities for improvement.

The analysis of patient‐centred outcomes revealed that approximately 56% of PHC users reported a positive experience with care. This level of satisfaction is consistent with findings from other countries in Central Asia and Eastern Europe, where patient‐reported satisfaction typically ranges between 50% and 70%, depending on service accessibility, continuity of care and provider communication. The moderate satisfaction levels observed in the included studies likely reflect the transitional nature of healthcare reforms in Kazakhstan, particularly the introduction of the compulsory social health insurance (CSHI) system. Studies by Shurenova et al. (2024a, 2024b) consistently noted that, while patients acknowledge improvements in service availability and organizational processes, gaps remain in areas such as consultation time, provider communication and waiting times. This pattern parallels trends reported in post‐Soviet healthcare systems undergoing similar reforms, where patient‐centredness often lags behind improvements in infrastructure and financing (Semenova et al. 2024).

Shaki et al. (2025) also highlighted the impact of the COVID‐19 pandemic on patient experiences. The pandemic strained PHC capacity, disrupted continuity of care, and increased reliance on remote consultations, which may have contributed to both positive and negative perceptions of care. Although some patients reported improved responsiveness due to digital tools and triage mechanisms, others noted reduced in‐person appointments and longer delays. These mixed experiences align with international literature documenting a global decline in patient satisfaction during the pandemic, especially in systems where PHC resources were already limited (Filip et al. 2022).

System‐level performance indicators exhibited even greater variability. The pooled estimate of 64% for structural and organizational domains should be interpreted with caution. Given the very high heterogeneity (*I* ^2^ = 98.4%) and substantial differences in outcome definitions and measurement approaches, this estimate does not represent a precise or definitive measure of system‐level performance. Rather, it provides a broad descriptive overview of available evidence and should be considered exploratory. In this context, statistical pooling may obscure meaningful differences between studies rather than provide a reliable summary estimate. However, the substantial heterogeneity observed (*I* ^2^ = 98.4%) indicates that system‐level performance is far from uniform. Studies such as Shaltynov et al. (2022) documented major geographic disparities, with rural regions experiencing significantly lower accessibility due to workforce shortages, uneven facility distribution and longer travel distances. Similar inequalities have been widely reported across Central Asia, where urban–rural divides remain a major determinant of PHC utilization and outcomes (Shaltynov et al. 2025).

Murat et al. (2024) further demonstrated that the COVID‐19 pandemic exposed systemic vulnerabilities related to workforce capacity, preparedness and service continuity. Although Kazakhstan implemented several measures to enhance PHC readiness, such as scaling telemedicine and strengthening triage mechanisms, these strategies had uneven uptake across regions. The heterogeneity in PHC effectiveness during the pandemic reflects broader patterns observed in middle‐income countries, where resource constraints and pre‐existing disparities influence the resilience of PHC systems (Pradhan et al. 2023).

Service design innovations, such as those evaluated by Masharipova et al. (2022), highlight the potential of nursing‐led PHC models to improve workflow efficiency and expand the scope of services. Their findings align with international evidence suggesting that redesigned service processes—particularly those emphasizing interdisciplinary care and expanded nursing roles—can enhance PHC capacity, reduce waiting times and improve patient experience (Abou Malham et al. 2020). However, such innovations remain limited in scale in Kazakhstan and require broader implementation supported by regulatory, organizational and workforce reforms.

The cluster‐randomized pilot trial by Verthein et al. (2022), which assessed alcohol screening and brief intervention coverage, provides important insights into the challenges of implementing preventive services within PHC. Despite demonstrating feasibility, the study reported modest screening uptake, mirroring global evidence showing that implementation of behavioural interventions in PHC often falls short due to time constraints, provider training gaps and competing workload demands.

The observed heterogeneity across studies may reflect structural and contextual differences within Kazakhstan's PHC system rather than methodological inconsistencies alone. Variability in financing mechanisms, provider availability, facility capacity, and urban–rural distribution contributes to wide disparities in both patient‐reported outcomes and measured performance indicators. Moreover, the divergence in outcome measures and study designs underscores the need for standardized PHC quality assessment tools, which remain insufficiently developed in many post‐Soviet health systems.

Despite these challenges, the findings also highlight several strengths of Kazakhstan's PHC sector. The moderate‐to‐high satisfaction levels and positive evaluations of service availability indicate that ongoing reforms, including CSHI implementation and digital health integration, may be contributing to improvements. Strengthening PHC governance, expanding preventive services, and scaling innovative care models could further enhance system performance.

The findings of this review are directly relevant to nursing practice because the pooled outcomes reflect domains in which PHC nurses have substantial responsibility, including patient communication, education, follow‐up, care coordination, preventive screening, chronic disease monitoring and continuity of care. The pooled patient satisfaction estimate of 56% suggests that patient experience in Kazakhstan's PHC system is moderate and may be improved through nursing‐sensitive interventions focused on communication, timely follow‐up, service navigation and continuity.

For PHC nurses, the findings support several practical actions. First, nurse‐led patient education and self‐management support should be strengthened, particularly for patients with chronic diseases and those requiring repeated contact with PHC services. Second, standardized triage, follow‐up and referral pathways may reduce waiting times and improve continuity of care. Third, reminder and recall systems led by nurses may improve preventive screening, vaccination and chronic disease monitoring. Fourth, telehealth follow‐up and community outreach may help improve access for rural and remote populations. Fifth, routine monitoring of nursing‐sensitive PHC indicators—such as communication quality, patient education, continuity, screening completion, response time and follow‐up adherence—may help PHC managers identify gaps in service delivery.

At the policy level, these findings support the need to expand nurse‐led models of care, strengthen PHC nursing workforce capacity, clarify nursing scope of practice and include nurses in quality improvement committees and CSHI‐related PHC reform planning. In this context, nurses should be viewed not only as service providers but also as key leaders in improving patient‐centred PHC quality in Kazakhstan.

### 4.1 Limitations

Despite the valuable insights generated by this review, several limitations should be considered. First, only eight studies were included, all from a single national context, which limits generalizability beyond Kazakhstan. In addition, geographic representation was uneven, with a predominance of studies conducted in urban settings, particularly Almaty, while rural and remote regions were underrepresented, potentially biassing pooled estimates.

Second, substantial heterogeneity was observed, especially for system‐level indicators (*I* ^2^ = 98.4%), reflecting both true differences in PHC organization and variability in outcome definitions, measurement approaches and study designs. This heterogeneity, combined with the small number of studies (*n* < 5 per meta‐analysis), limits the stability and interpretability of pooled estimates, which should therefore be considered exploratory.

Third, most included studies were cross‐sectional and some relied on convenience sampling, introducing potential selection bias and limiting causal inference and representativeness. Data limitations were also present, as some studies reported incomplete or non‐comparable outcomes, reducing the precision and consistency of the synthesis.

Finally, the review was restricted to English‐language publications and did not include grey literature, such as government reports or institutional data. This may have introduced language and publication bias and limited the comprehensiveness of the evidence base.

### 4.2 Future Directions

The findings of this review highlight several priorities for future research to strengthen evidence on primary healthcare (PHC) quality and accessibility in Kazakhstan. First, large, multi‐regional studies using standardized measurement tools are needed to improve comparability across settings, particularly by including underrepresented rural and remote populations through stratified sampling approaches.

Second, the development of validated, context‐specific instruments for assessing patient‐centred outcomes—such as satisfaction, communication, trust and continuity of care—is essential. Harmonized measurement frameworks aligned with WHO PHC performance recommendations would enable more reliable benchmarking and longitudinal monitoring of reforms, including the compulsory social health insurance (CSHI) system.

Future research should also integrate objective system‐level indicators (e.g., access, service utilization, screening coverage, digital health use and workforce capacity) alongside patient‐reported outcomes. Prospective cohort studies and intervention designs are particularly needed to evaluate the causal impact of organizational innovations, including nurse‐led care models, care coordination strategies, telemedicine and financing reforms.

In addition, the use of geospatial and administrative data should be expanded to better assess PHC accessibility, incorporating dynamic measures such as travel time, infrastructure availability and spatial distribution of vulnerable populations.

Finally, longitudinal studies are required to evaluate the long‐term effects of PHC reforms under the CSHI system, including their impact on equity, service coverage and system responsiveness. Complementary qualitative research may further elucidate barriers and facilitators of high‐quality PHC delivery.

## 5 Conclusion

This systematic review and meta‐analysis indicates that primary healthcare services in Kazakhstan show moderate patient satisfaction and variable system‐level performance. Although the findings should be interpreted cautiously because of the limited number of studies, high heterogeneity, and variability in outcome definitions, they identify important nursing‐sensitive gaps in communication, accessibility, continuity of care, service navigation, preventive screening and patient follow‐up.

For nursing practice and policy, the results highlight the need to strengthen nurse‐led care models, expand PHC nursing capacity, improve patient education and chronic disease follow‐up, integrate telehealth and rural outreach, and include nursing‐sensitive indicators in PHC quality monitoring. Strengthening the role of nurses in PHC quality improvement may contribute to better patient experience, improved access and more equitable primary healthcare delivery in Kazakhstan.

## Author Contributions

**Makhabbat Shurenova:** conceptualization, methodology, literature search, data curation, formal analysis, visualization, writing – original draft. **Amin Tamadon:** conceptualization, supervision, methodology, validation, writing – review and editing. **Nadiar M. Mussin:** investigation, interpretation, writing – review and editing. **Nigora G. Ashurova:** investigation, interpretation, writing – review and editing. **Ramazon Safarzoda Sharoffidin:** supervision, methodology, validation, writing – review and editing.

## Funding

This research received no external funding. All stages of the systematic review and meta‐analysis—including literature search, data extraction, quality appraisal and statistical synthesis—were conducted independently by the authors.

## Ethics Statement

This study analysed data extracted exclusively from published scientific articles. No primary or identifiable human data were obtained by the authors. Ethical approval for this research was not required; however, all included studies were checked to ensure that they reported obtaining approval from their respective institutional ethics committees and complied with international ethical standards for human subjects' research.

## Consent

The authors have nothing to report.

## Conflicts of Interest

The authors declare no conflicts of interest.

## Supporting information


**Table S1:** Extracted outcome data and harmonization approach.
**Table S2:** Full‐text articles excluded from the review with reasons.
**Table S3:** Risk‐of‐bias assessment.

## Data Availability

All data supporting the findings of this systematic review and meta‐analysis—including extracted datasets, search strategies, coding sheets and meta‐analysis scripts—are available upon reasonable request from the corresponding author. The authors deposited the final dataset and analysis files in an open‐access repository (OSF).
