# Supplementary material for: Quality of Primary Healthcare Services in Kazakhstan: A Systematic Review and Meta‐Analysis of Patient‐Centred Outcomes and Implications for Nursing Practice and Policy
Source: Nurs Open. 2026 Jun 17;13(6):e70648. doi: 10.1002/nop2.70648 (PMC13276004; doi:10.1002/nop2.70648)
Supplement: Supplementary file 1 — Table S1: Extracted outcome data and harmonization approach. Table S2: Full‐text articles excluded from the review with reasons. Table S3: Risk‐of‐bias assessment. [file NOP2-13-e70648-s001.docx]

Table S1. Extracted outcome data and harmonization approach

| Study | Outcome reported in paper | Extracted value (%) | Denominator (n) | Harmonization approach | References |
| --- | --- | --- | --- | --- | --- |
| Masharipova et al., 2022 | “Patient satisfaction with nursing communication (qualitative)” | NR | 10 | Not included in pooling; qualitative insights only | [1] |
| Murat et al., 2024 | “Organizational effectiveness indicators (correlation-based)” | NR | 10,459 | Converted qualitatively; not pooled due to heterogeneity | [2] |
| Orynbassarova, 2015 | “Satisfied with family doctor services” | 31% | 86 | Used as overall satisfaction indicator | [3] |
| Shaki et al., 2025 | “Satisfied” (overall patient satisfaction) | 45.2% | 1035 | Used as direct proportion of satisfied patients | [4] |
| Shaltynov et al., 2022 | “Accessibility index (Ai)” | NR | Population-based | Transformed conceptually; not pooled due to non-proportional metric | [5] |
| Shurenova et al., 2024a | “Completely satisfied with medical care” | 54.0% | 556 | Used as direct satisfaction proportion | [6] |
| Shurenova et al., 2024b | “Execution of PHC services / accessibility indicators” | 100% (plan execution) | NR | Converted to proportion of system-level performance | [7] |
| Verthein et al., 2022 | “Screening coverage / AUDIT-C positive” | 12.3% | 1148 | Used as system-level performance indicator | [8] |

Table S2. Full-text articles excluded from the review with reasons

| Author, Year | Title | Reason for Exclusion | References |
| --- | --- | --- | --- |
| Abdikadirova et al., 2025 | Patient Satisfaction with the Expanded Nurses Service in Primary Health Care | Different outcome definition (nurse-specific intervention; not general PHC system-level measure) | [9] |
| Abikulova et al., 2025 | Physical activity and alcohol intake among PHC workers | Population not relevant (healthcare workers; not PHC service quality) | [10] |
| Bazargaliyev et al., 2024 | Antidiabetic therapy in PHC | Disease-specific clinical management; not PHC quality indicators | [11] |
| Blushinova et al., 2025 | Induced abortion perceptions | Not related to PHC quality | [12] |
| Byltash et al., 2026 | GP dementia practices | Provider knowledge/practice study; no PHC quality outcomes | [13] |
| Dostanova et al., 2024 | Independent nursing consultations (qualitative) | Qualitative study; no extractable quantitative data | [14] |
| Iskakova et al., 2025 | HIV-related stigma in PHC | Focus on stigma; not PHC quality indicators | [15] |
| Kassymbekova et al., 2023 | HPV vaccine attitudes (protocol) | Study protocol; no results | [16] |
| Kozhekenova et al., 2025 | COVID-19 burden in PHC | Epidemiological outcomes; not PHC quality measures | [17] |
| Kurmanalina et al., 2025 | Autism awareness | Population awareness study; not PHC service quality | [18] |
| Nabirova et al., 2023 | Vaccine confidence among providers | Provider attitudes; not PHC system performance | [19] |
| Zhussupova et al., 2025 | Rational use of medicines framework | Organizational evaluation; not comparable PHC quality indicators | [20] |

Table S3. Risk-of-bias assessment

A. JBI assessment for observational/descriptive studies

| Study | Study design | JBI overall judgment | Main concerns | References |
| --- | --- | --- | --- | --- |
| Masharipova et al., 2022 | Descriptive experience / service design | Moderate risk | Small sample, qualitative/descriptive reporting, limited statistical analysis | [1] |
| Murat et al., 2024 | Cross-sectional survey | Moderate risk | Self-reported data, complex regression/factor analysis, limited external validation | [2] |
| Orynbassarova, 2015 | Pilot cross-sectional survey | High risk | Small convenience sample, no formal ethics review, limited representativeness | [3] |
| Shaki et al., 2025 | Cross-sectional survey | Low risk | Clear sampling, validated questionnaire, appropriate regression analysis | [3] |
| Shaltynov et al., 2022 | Descriptive GIS/secondary-data study | Moderate risk | Secondary/older census data, travel distance not travel time | [5] |
| Shurenova et al., 2024a | Cross-sectional observational study | Moderate risk | Limited to Almaty polyclinics, partial representativeness | [6] |
| Shurenova et al., 2024b | Retrospective secondary-data study | Moderate risk | Administrative data, limited detail on service quality outcomes | [7] |

B. RoB 2 assessment for randomized trial

| Study | Tool | Overall judgment | Main concerns | Reference |
| --- | --- | --- | --- | --- |
| Verthein et al., 2022 | Cochrane RoB 2, cluster randomized trial | Some concerns | One PHCU dropped out after randomization; low follow-up among AUDIT-C positive patients; pilot feasibility design | [8] |

**References**

1. Masharipova AV, Derbissalina GA, Zhunussova DK, Nagashybek G, Amangeldiyeva D. Experience in using the service design in the development of nursing services in the republic of Kazakhstan. The New Armenian Medical Journal. 2022;16(3):43-8. doi:10.56936/18290825-2022.16.3-43

2. Murat A, Otargaliyeva D, Tabarov A, Saliev T, Fakhradiev I. Effectiveness of primary health care in the Republic of Kazakhstan during the COVID-19 pandemic and factors affecting it. Disaster and Emergency Medicine Journal. 2024;9(4):208-25. doi:10.5603/demj.101057

3. Orynbassarova D. Family Medicine as a Model of Primary Health Services Delivery: A Pilot Study in Almaty, Kazakhstan. Cent Asian J Glob Health. 2015;4(1):209. doi:10.5195/cajgh.2015.209

4. Shaki D, Aimbetova G, Baysugurova V, Kanushina M, Chegebayeva A, Arailym M, et al. Level of Patient Satisfaction with Quality of Primary Healthcare in Almaty During COVID-19 Pandemic. Int J Environ Res Public Health. 2025;22(5):804. doi:10.3390/ijerph22050804

5. Shaltynov A, Rocha J, Jamedinova U, Myssayev A. Assessment of primary healthcare accessibility and inequality in north-eastern Kazakhstan. Geospat Health. 2022;17(1). doi:10.4081/gh.2022.1046

6. Shurenova M, Kurakbayev K, Abildaev T, Tazhiyeva A. Availability and quality of primary health care in the compulsory health insurance system in Kazakhstan. Med Glas (Zenica). 2024;21(1):159-65. doi:10.17392/1675-23

7. Shurenova M, Kurakbayev K, Abildaev T, Tazhiyeva A. Primary healthcare services' accessibility and quality under compulsory social health insurance in Kazakhstan. Front Public Health. 2024;12:1418367. doi:10.3389/fpubh.2024.1418367

8. Verthein U, Lahusen H, Martens MS, Prilutskaya M, Yussopov O, Kaliyeva Z, et al. Alcohol Screening and Brief Intervention in Primary Health Care in Kazakhstan-Results of a Cluster Randomised Pilot Study. Int J Public Health. 2022;67:1604803. doi:10.3389/ijph.2022.1604803

9. Abdikadirova I, Yermukhanova L, Blazeviciene A, Dostanova Z, Baigozhina Z, Taushanova M, et al. Patient Satisfaction with the Expanded Nurses Service in Primary Health Care: Evidence from Kazakhstan. Healthcare (Basel). 2025;13(24). doi:10.3390/healthcare13243314

10. Abikulova A, Qumar AB, Seiduanova L, Kuttybayev A, Davletov D, Jussipbekova B, et al. Physical activity and alcohol intake among primary health care workers in Almaty, Kazakhstan: a cross-sectional study. BMC Public Health. 2025;25(1):4220. doi:10.1186/s12889-025-25265-9

11. Bazargaliyev Y, Tleumagamabetova B, Kudabayeva K, Kosmuratova R. Analysis of Antidiabetic Therapy for Type 2 Diabetes in Primary Health Care (Western Kazakhstan). Georgian Med News. 2024(348):22-7.

12. Blushinova A, Orazalina A, Shalgumbayeva G. Induced Abortion in Kazakhstan: Women's Perceptions and Experiences Based on Cross-Sectional Study. Georgian Med News. 2025(364-365):280-8.

13. Byltash S, Brimzhanova M, Yeshmanova A, Akhmetzhan A, Kosherbayeva L. General practitioners' practices and training needs in dementia care: a comparison of urban and rural areas in Kazakhstan. Aging Ment Health. 2026;30(2):277-86. doi:10.1080/13607863.2025.2564720

14. Dostanova Z, Yermukhanova L, Blazeviciene A, Baigozhina Z, Taushanova M, Abdikadirova I, et al. Perception and Experience of Independent Consultations in Primary Healthcare among Registered Nurses in Kazakhstan: A Qualitative Study. Healthcare (Basel). 2024;12(15). doi:10.3390/healthcare12151461

15. Iskakova B, King EJ, Yucel RM, DeHovitz J, Nugmanova Z. "I think they are infected because of their ignorance and lack of responsibility": A mixed-methods study on HIV-related stigma in the healthcare system in Kazakhstan. PLoS One. 2025;20(9):e0331201. doi:10.1371/journal.pone.0331201

16. Kassymbekova F, Zhetpisbayeva I, Tcoy E, Dyussenov R, Davletov K, Rommel A, et al. Exploring HPV vaccine knowledge, attitudes, barriers and information sources among parents, health professionals and teachers in Kazakhstan: a mixed-methods study protocol. BMJ Open. 2023;13(9):e074097. doi:10.1136/bmjopen-2023-074097

17. Kozhekenova N, Moiynbayeva S, Jeremic D, Dinic M, Semenov P, Nurgaliyeva Z, et al. The burden of COVID-19 in primary care of Almaty, Kazakhstan, 2021-2022. Sci Rep. 2025;15(1):5186. doi:10.1038/s41598-025-89707-5

18. Kurmanalina S, Samambayeva A, Akhtayeva N, Kozhageldiyeva L, Kosherbayeva L. Awareness of Autism Spectrum Disorder Among Population of Kazakhstan. J Autism Dev Disord. 2025;55(7):2566-72. doi:10.1007/s10803-024-06350-1

19. Nabirova D, Horth R, Kassabekova L, Henderson A, Yesmagambetova A, Alaverdyan S, et al. Factors associated with COVID-19 vaccine confidence among primary care providers in Kazakhstan, March-April 2021. Front Public Health. 2023;11:1245750. doi:10.3389/fpubh.2023.1245750

20. Zhussupova G, Aiypkhanova A, Zhaldybayeva S, Satmbekova D, Akhayeva T, Kaliyeva S. Evaluation of a national framework for rational use of medicines in Kazakhstan and its role in improving medicine use practices at the organizational and national levels. BMC Health Serv Res. 2025;25(1):49. doi:10.1186/s12913-024-12172-9
